# Correction

**DOI:** 10.1080/24740527.2025.2533718

**Published:** 2025-07-25

**Article title**: Neuroethical issues in adopting brain imaging for personalized chronic pain management: Attitudes of people with lived experience of chronic pain

**Authors**: Karen Davis, Monica de Oliveira, Ariana Besik, and Daniel Z. Buchman

**Journal**: *Canadian Journal of Pain*

**Bibliometrics**: Volume 08, Number 02, pages 01 - 11

**DOI**: http://dx.doi.org/ 10.1080/24740527.2024.2425596

The article titled “ Neuroethical issues in adopting brain imaging for personalized chronic pain management: Attitudes of people with lived experience of chronic pain” by Karen Davis, Monica de Oliveira, Ariana Besik, and Daniel Z. Buchman which was published in *Canadian Journal of Pain* Volume 8, Issue 2, was incorrectly assigned to this issue.

This article should have appeared in Volume 9, Issue 1 of *Canadian Journal of Pain*. This error does not impact the academic content of the article.

